# Tri-Ortho-Cresyl Phosphate Inhibits Proliferation of Mouse Germ Cells by Activating Endoplasmic Reticulum Stress and Suppressing NTE Activity

**DOI:** 10.3390/toxics14040275

**Published:** 2026-03-25

**Authors:** Dan Yang, Di Zhang, Xiao-Hua Song, Xiang-Dong Li

**Affiliations:** 1 State Key Laboratory of Animal Biodiversity Conservation and Integrated Pest Management, Institute of Zoology, Chinese Academy of Sciences, 1-5 Beichenxilu Road, Beijing 100101, China; 18754883207@163.com (D.Z.); monster1song@163.com (X.-H.S.); lixd@ioz.ac.cn (X.-D.L.); 2 University of Chinese Academy of Sciences, Beijing 100049, China

**Keywords:** TOCP, GC-1 spg cells, proliferation, mitosis, ER stress, NTE

## Abstract

Tri-o-cresyl phosphate (TOCP) is widely used as a plasticizer, flame retardant, and lubricant additive, but has been reported to impair spermatogenesis. However, how TOCP affects spermatogenesis remains unclear. Therefore, the objective of this study is to investigate the underlying mechanism by which TOCP disrupts spermatogenesis. In order to achieve this, adult male mice were orally administered TOCP at doses of 0, 200, or 400 mg/kg for two weeks, and we found that TOCP exposure reduced the number of germ cells and decreased sperm density. Moreover, the numbers of PCNA-positive cells and phospho-histone H3 (Ser10)-positive cells in mouse testicular tissues were significantly decreased following TOCP treatment, indicating that germ cell proliferation may be impaired. In addition, TOCP did not affect the protein expression of neuropathy target esterase (NTE) in testicular tissues but markedly inhibited its enzymatic activity (by approximately 30% relative to the control level). In vitro experiments further demonstrate that TOCP suppressed cell proliferation and mitotic progression in mouse GC-1 spg cells and excessively activated endoplasmic reticulum (ER) stress. Treatment with 4-phenylbutyric acid (4-PBA), an ER stress inhibitor, partially reversed the TOCP-induced inhibition of cell proliferation and mitosis. Furthermore, TOCP inhibited NTE activity in GC-1 spg cells, and NTE knockdown produced a phenotype similar to that observed after TOCP exposure, characterized by suppressed cell proliferation and mitotic progression. Surprisingly, ER stress was not activated in GC-1 spg cells following NTE knockdown. Collectively, these findings suggest that TOCP may impair spermatogenesis by inhibiting the proliferation and mitotic progression of mouse germ cells, potentially through mechanisms involving excessive activation of ER stress or suppression of NTE activity.

## 1. Introduction

The discontinuation of polybrominated diphenyl ethers (PBDEs) has resulted in an increase in the manufacture and application of substitute flame retardants, such as organophosphate flame retardants (OPFRs) [1,2]. Tri-o-cresyl phosphate (TOCP), an organophosphorus compound, is utilized as a flame retardant and plasticizer in industrial products [3]. TOCP can, however, accumulate in the brain, testes, liver, kidneys, and plasma [4]. Mounting evidence indicates that TOCP exerts multiple toxicities such as hepatotoxicity [5,6], neurotoxicity [7,8] and reproductive toxicity. Notably, TOCP has been detected in the blood of pregnant women and in umbilical cord samples, posing potential risks to both mothers and their offspring due to its slow elimination from the body [9]. It has been established that TOCP disrupts follicular development and steroidogenesis, ultimately leading to ovarian dysfunction or failure [10,11,12]. In addition, TOCP has been reported to induce testicular damage and reduce sperm count and motility [13,14,15,16]. However, the precise mechanisms underlying its testicular and reproductive toxicity remain incompletely understood.

TOCP induces a well-characterized neurotoxic condition known as organophosphate-induced delayed neurotoxicity (OPIDN), primarily through inhibition of neuropathy target esterase (NTE) [17,18,19,20]. NTE is a serine hydrolase with phospholipase B activity that catalyzes the hydrolysis of phosphatidylcholine to generate glycerophosphocholine and free fatty acids [21,22,23]. NTE is predominantly localized to the endoplasmic reticulum and is widely expressed in multiple tissues, including the testes, lungs, spleen, brain, and kidneys. It plays a critical role in embryonic development and cellular homeostasis [24]. Genetic mutations or functional alterations in NTE have been associated with various neurodegenerative disorders, some of which are accompanied by clinical manifestations such as hypogonadism [25].

The endoplasmic reticulum (ER) plays a vital role in managing intracellular calcium levels, lipid production, and the proper folding of proteins, all of which are essential to maintaining homeostasis. ER stress arises from fluctuations in luminal Ca^2+^ levels, alterations in redox conditions, an increase in misfolded proteins, or excessive protein accumulation [26]. To counteract ER stress, the unfolded protein response (UPR) is initiated, aiming to restore proper protein management when misfolded proteins are detected within the ER lumen [27]. During ER stress, the activation of UPR helps to minimize the buildup of misfolded proteins by expanding the ER membrane, inhibiting protein synthesis, and reducing the influx of proteins into the ER [27,28]. Nevertheless, if the UPR is unable to alleviate ER stress, it may lead to cell death through mechanisms such as CHOP [26].

This study focuses on exploring the toxic effects of TOCP on the male reproductive system and understanding the contributions of ER stress and NTE to the reproductive toxicity caused by TOCP.

## 2. Materials and Methods

### 2.1. Chemicals

Tri-ortho-cresyl phosphate (TOCP, CAS: 78-30-8, 96% purity) was purchased from Acros Organics (Geel, Belgium). The 4-phenylbutyrate and thapsigargin were purchased from Shanghai Yuanye Bio-Technology Co., Ltd. (Shanghai, China). The other chemicals used are detailed below.

### 2.2. Antibodies

Antibodies against GRP78 (#3177), CHOP (#2895), BrdU (#5292), Phospho-H3 (Ser10) (#53348), and PCNA (D3H8P) were purchased from Cell Signaling Technology (Boston, MA, USA). Antibody against NTE (ab228683) was purchased from Abcam (Cambridge, UK). Antibodies against β-actin and GAPDH were purchased from Cowin Biotechnology (Beijing, China).

### 2.3. Animals and Treatment

Eight-week-old male C57BL/6 mice were acquired from SPF (Beijing) Biotechnology Co., Ltd. (Beijing, China), and acclimated in our breeding facility. The animals were housed in cages with ad libitum access to food and water under controlled environmental conditions (temperature 20–26 °C) and a 12 h light/dark cycle. After a period of acclimatization, the mice were randomly assigned to three groups, with 15 animals per group.

The mice received oral doses of TOCP at varying concentrations (200 and 400 mg/kg of body weight per day, dissolved in 100% DMSO and administered to mice by gavage at a volume of 10 mL/kg), while those in the control group were administered the same volume of the solvent. All animal experiments complied with the relevant Chinese laws and regulations. The experimental protocols involving animals were reviewed and approved by the Animal and Medical Ethics Committee of the Institute of Zoology, Chinese Academy of Sciences. The investigators responsible for animal dosing and outcome assessment were blinded to the group allocation until the statistical analysis was completed. The sample size (*n* = 15 per group) was chosen based on the previously published literature in the field and the experiment design, rather than on a formal power analysis.

### 2.4. Histopathology

For histological analysis, the testes of four mice and cauda epididymis were randomly selected for observation. The testes and epididymis samples, fixed with paraformaldehyde, underwent dehydration through a series of ethanol concentrations, were cleared using xylene, and were subsequently embedded in paraffin wax. Using an RM2135 rotary microtome (Lecia, Wetzlar, Germany), tissue blocks were sliced into 7 μm sections and stained with hematoxylin and eosin (Solarbio, Beijing, China). The slides were then covered with a neutral balsam and analyzed under an Olympus IX71 microscope (Tokyo, Japan). Testes from four mice per group were each used to prepare five consecutive sections; at least three sections were randomly selected from each mouse. Then, at least five images were randomly captured from each section. The images were independently evaluated by two professional pathologists. The animal was the statistical unit, and the multiple measurements per animal were quantified and averaged to a value.

### 2.5. Sperm Count Analysis

Both epididymes from each animal were separated, trimmed free of fat, and then placed on the filter paper to remove any liquid. The dissected epididymes were weighed and stored in 2 mL of prewarmed phosphate-buffered saline (PBS) at 37 °C. Each epididymis was repeatedly punctured with a sterile needle and placed in an incubator at 37 °C for 30 min to enable the sperm to swim out. After incubating part of the sperm suspension at 65 °C for 10 min, 10 μL of suspension was taken and assessed using a hemocytometer. The total number of sperm was calculated as the volume of sperm medium (2 mL of PBS) multiplied by the sperm concentration (sperm/mL) and then divided by epididymal weight (g). Four mice per group were used to prepare samples. The animal was the statistical unit, the multiple measurements per animal were quantified and averaged to a value.

### 2.6. Immunohistochemistry

Testicular paraffin sections were treated to remove wax using xylene and varying concentrations of ethanol, followed by antigen retrieval via microwave heating. To inhibit endogenous peroxidase activity, the sections were exposed to 3% hydrogen peroxide for 10 min. After being rinsed three times with deionized water, the sections were incubated in 5% BSA for 1 h. Primary antibodies targeting PCNA and phosphor-H3 (Ser 10) were then applied to the sections and left overnight at 4 °C. The secondary antibody was incubated at 37 °C for 1 h, and diaminobenzidine was utilized to visualize the antibody–antigen complexes. Finally, hematoxylin was used for counterstaining, and the slides were dehydrated using a series of ethanol concentrations before being sealed with neutral gum. Testes from four mice per group were each used to prepare five consecutive sections; at least three sections were randomly selected from each mouse. The animal was the statistical unit, and the multiple measurements per animal were quantified and averaged to a value.

### 2.7. Cell Culture and Treatment

GC-1 spg cells were acquired from iCell (Xiao Fan Technology Co., Ltd., Guangzhou, China). The cells were cultured in Dulbecco’s modified Eagle’s medium (DMEM) (Gibco, Grand Island, NY, USA) supplemented with 10% fetal bovine serum, 100 IU/mL of penicillin, and 100 μg/mL of streptomycin. The cultures were maintained at 37 °C in a humidified incubator with 5% CO_2_. The cells were kept in the logarithmic growth phase and were sub-cultured every two days.

GC-1 spg cells were seeded in a 96-well plate at a density of 1 × 10^4^ cells per well. They were exposed to varying concentrations of TOCP (0 μM, 62.5 μM, 125 μM, 250 μM, 500 μM, and 1000 μM), which was dissolved in DMSO, for a duration of 24 h. Two hours prior to the end of the treatment, MTT (0.5 mg/mL) was added to the cells, followed by the addition of DMSO for 15 min. The viability of the cells was assessed by measuring absorbance at 570 nm using a spectrophotometer.

The cells were treated with TOCP (62.5 μM, 125 μM, and 250 μM) for 24 h, while the cells in the vehicle control group were given the equivalent solvent. Following this, assessments were made regarding cell growth, mitotic activity, levels of free calcium ions within the cells, and the expression of specific proteins. After a 2 h pretreatment with 2 mM 4-PBA, an inhibitor of endoplasmic reticulum stress, the cells were subsequently treated with 250 μM TOCP for another 24 h, while the cells in the positive control group were treated with 1 μM thapsigargin (an endoplasmic reticulum stress activator) for 24 h. Immunofluorescence techniques were then employed to evaluate cell proliferation and mitosis.

### 2.8. Cell Proliferation Analysis

The cells were treated with DMEM containing BrdU dye at a final concentration of 0.03 mg/mL for 4 h. After this period, the cell slides were removed and rinsed twice with PBS for 5 min each. The cells were then fixed using 70% ethanol for 5 min at room temperature and subsequently incubated in 1.5 mol/L hydrochloric acid for 30 min. Following this, a blocking solution of 5% BSA was applied for 1 h. The cells were then incubated overnight at 4 °C with a diluted BrdU antibody and, afterward, a diluted fluorescent secondary antibody was added in the dark for 1 h. Imaging was conducted using confocal laser scanning microscopes (STELLARIS 5, Lecia, Wetzlar, Germany), and the BrdU fluorescence intensity was quantitatively analyzed using ImageJ (Java 1.8.0). Three cell slides were prepared for each group, and at least five images were taken for each cell slide.

### 2.9. Cell Mitosis Analysis

The cell slides were taken out of the 24-well plate and placed in a humid box. The cells underwent fixation using 4% paraformaldehyde for a duration of 15 min. To permeabilize the cultures, a solution of 0.5% Triton X-100 in phosphate-buffered saline was applied for 10 min, followed by a 1 h incubation with 5% BSA. After an overnight incubation at 4 °C with the Phospho-Histone 3 (Ser10) antibody, the cells were treated with a species-specific secondary antibody for 1 h. Intracellular fluorescence was examined using confocal laser scanning microscopy (STELLARIS 5, Leica, Wetzlar, Germany). The fluorescence intensity of Phospho-H3 (Ser 10) was quantitatively analyzed using ImageJ (Java 1.8.0). Three cell slides were prepared for each group, and at least five images were taken for each cell slide.

### 2.10. [Ca^2+^]_i_ Assay

The cultured cells were digested with pancreatic enzymes and collected in centrifuge tubes. After washing the cells twice with PBS, the cell concentration was adjusted to 2 × 10^6^/mL using serum-free DMEM. The cell suspension was incubated with a 5 μM Fura-2/AM fluorescent probe at 37 °C for 40 min while protected from light. After incubation, the cultures were washed twice with serum-free DMEM and resuspended in PBS prior to being kept at room temperature for 20 min in the dark. The microplate reader (SpectraMax i3, Molecular Devices, San Jose, CA, USA) was used to detect the fluorescence intensity at the constant emission wavelength of 510 nm, and the excitation wavelengths of 340 nm and 380 nm, respectively. Subsequently, the fluorescence intensity at these wavelengths was measured with the addition of 2.5% Triton X-100, followed by the detection at the above wavelengths after the addition of 250 mmol/L EGTA. The concentration of free calcium ions was found according to the formula ${\left[{\mathrm{Ca}}^{2+}\right]}_{i}=\mathrm{kd}\times \frac{F_{0}}{F_{s}}\times \frac{R-R_{\min}}{R_{\max}-R}$ (in which *kd* = 224 nmol/L; *F_0_* represents the fluorescence intensity upon excitation at 380 nm in the absence of calcium ion; *F_s_* represents the fluorescence intensity upon excitation at 380 nm under calcium ions saturation conditions; *R* represents the fluorescence ratio F340/F380; *R_max_* represents the fluorescence ratio after adding only 2.5% Triton X-100; and *R_min_* represents the fluorescence ratio after adding 2.5% Triton X-100 and 250 mmol/L EGTA).

### 2.11. Western Blotting Analysis

Protein extracts were prepared by homogenizing cultured cells in RIPA buffer. After centrifugation at 1000× *g* for 15 min to clarify the lysates, the protein content was measured via the Bradford method [29]. Following denaturation at 100 °C for 10 min, the protein samples were resolved by 10% SDS-PAGE and transferred to PVDF membranes. The membranes were blocked with 5% skim milk or bovine albumin V in TBST and incubated with primary antibodies overnight at 4 °C. After three washes with TBST, the membranes were incubated with HRP-conjugated secondary antibodies for 2 h. Immunoreactive bands were detected using enhanced chemiluminescence (ECL) reagents (Beyotime Biotechnology, Shanghai, China) and captured by a Micro-Chemi 4.2 imaging system (DNR Bio-Imaging Systems Ltd., Jerusalem, Israel). Densitometric analysis was performed using ImageJ software (Java 1.8.0), with protein expression normalized to β-actin or GAPDH.

### 2.12. Reverse Transcription and Quantitative Real-Time PCR

Testicular total RNA was isolated using Trizol (ThermoFisher, Waltham, MA, USA). Complementary DNAs were synthesized from 0.5 μg RNA using UEIris II RT MasterMix (BioDee Biotechnology Co., Ltd., Beijing, China). qPCR analysis was conducted with SYBR Green I Master Mix (Tsingke Biotechnology Co., Ltd., Beijing, China) as per the supplier’s guidelines. Relative quantification of target mRNA was performed using the 2^−ΔΔCt^ method, with *β-actin* as the reference gene. The specific primers used in this study are summarized in Table 1. Testes from three mice were used to prepare samples. The animal was the statistical unit, and the multiple measurements per animal were quantified and averaged to a value.

### 2.13. RNA Interference and Generation of Stable Cell Lines

For lentivirus production, shRNA sequences (Table 2) were cloned into the pLKO.1-mcherry vector. HEK293T cells were then co-transfected with these plasmids, along with the packaging vectors psPAX2 and pMD2. G. Culture medium containing lentiviral particles was collected 48 h post transfection and used to infect GC-1 spg cells. Infected cells were selected with 2 μg/mL puromycin to obtain stably transfected populations.

### 2.14. NTE Activity Analysis

NTE activity was determined from the difference in phenol liberated from the samples exposed to paraoxon and from those with both paraoxon and mipafox hydrolysis of phenyl valerate by colorimetric assay according to Johnson [30]. Based on the previous reference [31], an improved method for NTE activity determination was developed in our laboratory, in which testes or GC-1 spg cells were homogenized in TE buffer (50 mM Tris-HCL, 0.2 mM EDTA, pH 8.0) and centrifuged at 100× *g* for 5 min. The following four reaction systems were set up: (I) the supernatant added with 400 μM paraoxon and 200 μM mipafox; (II) the supernatant added with 400 μM paraoxon and TE buffer; (III) 400 μM paraoxon and 200 μM mipafox; and (IV) TE buffer. The reaction systems were incubated at 37 °C for 30 min; then, the substrate solution (a mixture of 1 volume of 15 mg/mL phenyl valerate and 30 volumes of 0.03% Triton X-100 in TE buffer) was added to reaction systems (I), (II), and (III), while 0.03% Triton X-100 in TE buffer was added to reaction system (IV). After accurate incubation at 37 °C for 30 min, the reaction was stopped with termination solution (3.32% SDS/0.025% 4-aminoantipyrine in TE buffer). Adding 0.4% potassium ferricyanide into the TE buffer for 5 min was followed by measuring the absorbance at 486 nm by spectrophotometer. The protein content was determined by the method of Bradford [29].

### 2.15. Statistical Analysis

Statistical data analysis was performed using GraphPad Prism 8.0.2 (GraphPad Software, La Jolla, CA, USA). Data are expressed as the mean ± SEM. Statistical comparisons among multiple groups were performed using one-way ANOVA, followed by Dunnett’s multiple comparisons test. A *p*-value less than 0.05 was considered statistically significant. The animal was the statistical unit.

## 3. Results

### 3.1. TOCP Inhibits the Proliferation of Germ Cells in the Seminiferous Tubules of Mice Testes

**Figure 1:**
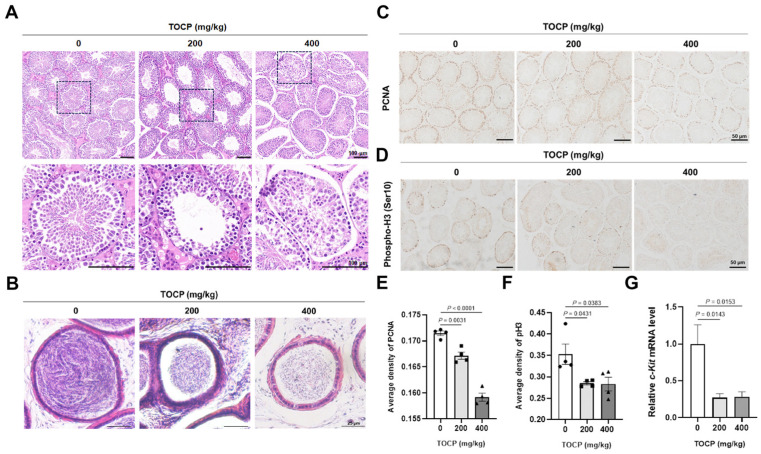
TOCP inhibits proliferation of germ cells in mice testis tissue. Adult male mice were treated with 0, 200, 400 mg/kg TOCP for 14 days. The structure of testes (**A**) and the sperm count in cauda epididymes (**B**) were observed by H&E staining (*n* = 4; for each animal, three sections and at least five fields per section were quantified and averaged). PCNA (**C**,**E**) and phospho-H3 (Ser 10) (**D**,**F**) expression was observed by immunohistochemistry staining (*n* = 4, for each animal, three sections and at least five fields per section were quantified and averaged). (**G**) The mRNA level of *c-Kit* in testis tissue was determined by qPCR (*n* = 3). Data are expressed as mean ± SEM. The animal was the statistical unit, and each dot represents one mouse. Statistical significances were determined by using one-way ANOVA followed by Dunnett’s multiple comparisons test. *p* < 0.05 indicates a significant difference between groups.

To investigate whether TOCP can cause testicular damage, adult male mice were orally administrated with 0, 200, 400 mg/kg TOCP for 14 days. Compared to the control group, the TOCP-exposed mice exhibited a significantly lower body weight and epididymis weight, but showed no significant change in testis weight (Figure S1), suggesting that the administration of 400 mg/kg TOCP may have induced systemic toxicity. As shown in Figure 1A, the structure of the seminiferous tubules was disordered and the number of germ cells was significantly decreased in the TOCP-treated testis tissue. Furthermore, TOCP could also reduce the sperm count of the cauda epididymis (Figure 1B and Figure S2), indicating that TOCP might inhibit spermatogenesis. Subsequently, we found that there was a decrease in the expression of PCNA in the TOCP-treated seminiferous tubule, implying that TOCP can inhibit proliferation of mouse germ cells (Figure 1C,E). Meanwhile, the expression of phospho-H3 (Ser 10), a mitotic activity marker, was also significantly decreased in the TOCP-treated testis tissue (Figure 1D,F), as was the mRNA level of *c-Kit* was significantly decreased in the TOCP-exposed testis tissue (Figure 1G). These findings indicate that TOCP inhibits proliferation of mouse germ cells, thereby causing spermatogenesis arrest.

### 3.2. TOCP Inhibits Cell Proliferation and Mitosis of Mouse GC-1 spg Cells

**Figure 2:**
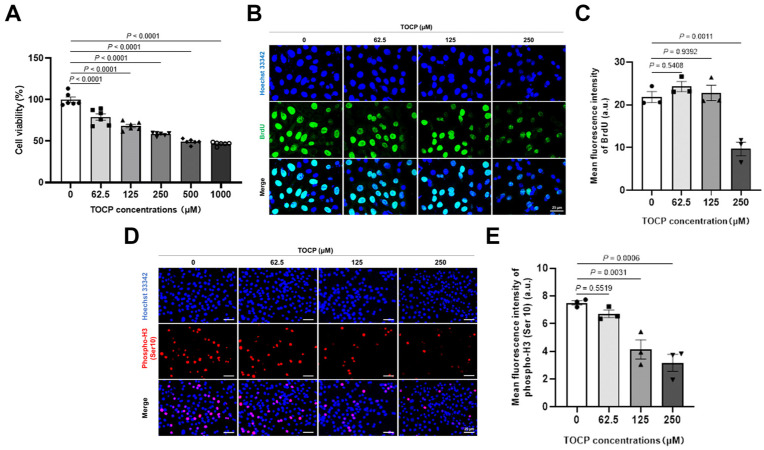
TOCP inhibits proliferation and mitosis of mouse GC-1 spg cells. (**A**) Cell viability was measured by MTT assay after mouse GC-1 spg cells were treated with 0, 62.5, 125, 250, 500, and 1000 μM TOCP for 24 h (*n* = 6). BrdU (**B**,**C**) and phospho-H3 (Ser 10) (**D**,**E**) were stained after mouse GC-1 spg cells were treated with 0, 62.5, 125, and 250 μM TOCP for 24 h. Data are presented as mean ± SEM (*n* = 3; for each replicate in per group, three slides were randomly selected, and at least five random fields were examined per cell slide). Each replicate was the statistical unit, and each dot corresponds to one replicate. Statistical significances were determined by using one-way ANOVA followed by Dunnett’s multiple comparisons test. *p* < 0.05 indicates a significant difference between groups.

In order to further confirm the effect of TOCP on proliferation and mitosis of mouse spermatogonial cells, mouse GC-1 spg cells, a mouse-derived spermatogonial cell line, were treated with 0, 62.5, 125, 250, 500, and 1000 μM TOCP for 24 h. As can be seen from Figure 2A, TOCP inhibited viability of GC-1 spg cells in a concentration-dependent manner. We selected 0, 62.5, 125, and 250 μM TOCP for subsequent experiments. Next, the expression of BrdU was found to have decreased in the 1000 μM TOCP- treated cells (Figure 2B,C), and the expression of phospho-H3 (Ser 10) was found to have decreased in the TOCP-treated cells (Figure 2D,E). These results indicate that TOCP can inhibit proliferation and mitosis of GC-1 spg cells.

### 3.3. TOCP Induces Endoplasmic Reticulum (ER) Stress in Mouse GC-1 spg Cells

**Figure 3:**
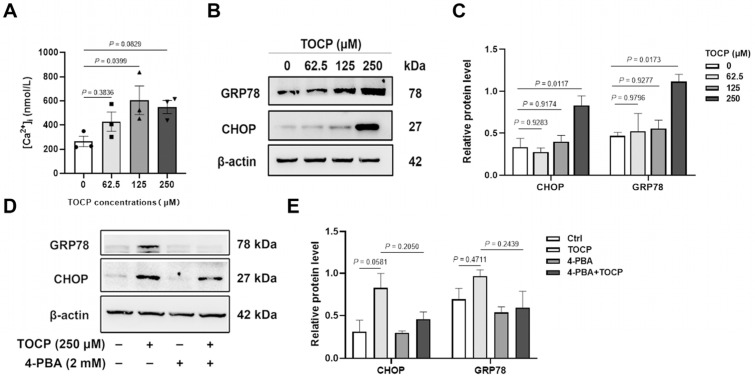
TOCP induces endoplasmic reticulum stress of mouse GC-1 spg cells. The concentration of intracellular free calcium (**A**) and the protein levels of CHOP and GRP78 (**B**,**C**) were detected after mouse GC-1 spg cells were treated with 0, 62.5, 125, and 250 μM TOCP for 24 h. (**D**,**E**) The expression of CHOP and GRP78 was determined after mouse GC-1 spg cells were treated with 250 μM TOCP for 24 h in the presence or absence of 2 mM 4-PBA. Data are presented as mean ± SEM (*n* = 3). Statistical significances were determined by using one-way ANOVA followed by Dunnett’s multiple comparisons test. *p* < 0.05 indicates a significant difference between groups.

To investigate the potential mechanism of TOCP-induced inhibition of proliferation and mitosis in mouse spermatogonial cells, we first detected the concentration of intracellular calcium ion, which is involved in cell proliferation, differentiation, endoplasmic reticulum (ER) stress, and muscle contraction. An increase in cytosolic free calcium concentration induces ER stress, and the reciprocal enhancement between elevated intracellular Ca^2+^ levels and ER stress activation constitutes a positive feedback loop [32]. As shown in Figure 3A, the concentration of intracellular free calcium ion was increased in the TOCP-treated cells compared with the control cells. Subsequently, the expression of ER stress biomarkers such as GRP78 and CHOP was increased in the TOCP-treated cells, while 2 mM 4-phenylbutyrate (4-PBA) attenuated TOCP-induced upregulation of GRP78 and CHOP in the cells (Figure 3B–E). These results suggest that TOCP can induce endoplasmic reticulum stress in GC-1 spg cells.

### 3.4. TOCP-Inhibited Proliferation and Mitosis of Mouse GC-1 spg Cells Were Associated with ER Stress

**Figure 4:**
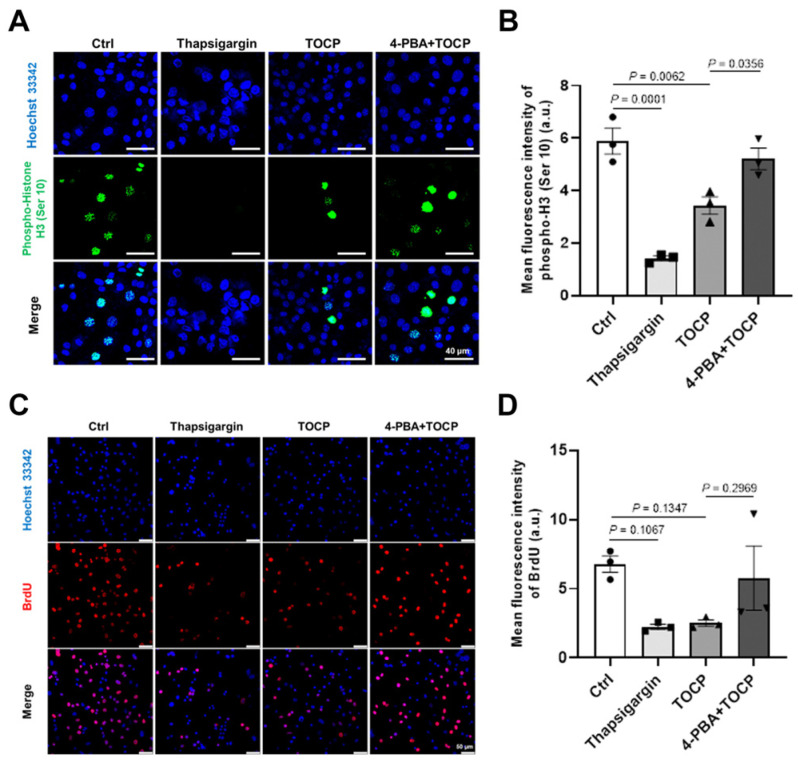
TOCP inhibits proliferation and mitosis of mouse GC-1 spg cells via the activation of endoplasmic reticulum stress. Cell mitosis (**A**,**B**) and cell proliferation (**C**,**D**) were observed by phosphor-H3 (Ser 10) and BrdU immunofluorescence staining, respectively, after mouse GC-1 spg cells were respectively treated with, respectively, DMSO, 250 μM TOCP, 1 μM thapsigargin or 2 mM 4-PBA (pretreated for 2 h) plus 250 μM TOCP for 24 h. Data are presented as mean ± SEM (*n* = 3, for each replicate in per group, three slides were randomly selected, and at least five random fields were examined per cell slide). Each replicate was the statistical unit, and each dot corresponds to one replicate. Statistical significances were determined by using one-way ANOVA followed by Dunnett’s multiple comparisons test. *p* < 0.05 indicates a significant difference between groups.

Next, we wondered whether ER stress was involved in the TOCP-induced inhibition of proliferation and mitosis of mouse spermatogonial cells. As shown in Figure 4, TOCP significantly decreased the expression of phospho-H3 (Ser 10), and decreased the expression of BrdU in mouse GC-1 spg cells, while 4-PBA alleviated the decrease caused by TOCP. These results suggest that TOCP-inhibited proliferation and mitosis of mouse GC-1 spg cells were associated with ER stress markers.

### 3.5. NTE Is Involved in TOCP-Induced Inhibition of Proliferation and Mitosis of Mouse GC-1 spg Cells

**Figure 5:**
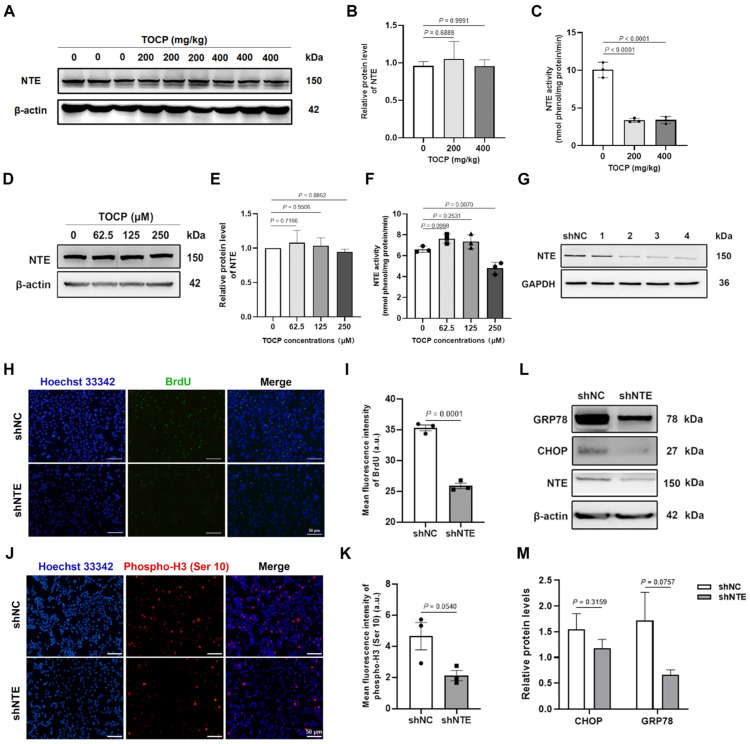
NTE is involved in TOCP-induced inhibition of proliferation and mitosis of mouse GC-1 spg cells. The NTE protein levels (**A**,**B**) and activity (**C**) in testis tissue were detected after adult male mice were treated with 0, 200, and 400 mg/kg TOCP for 14 days (*n* = 3, the animal was the statistical unit). The NTE protein level (**D**,**E**) and NTE activity (**F**) were determined after mouse GC-1 spg cells were treated with 0, 62.5, 125, and 250 μM TOCP for 24 h (*n* = 3). The NTE protein level (**G**), the staining of BrdU ((**H**,**I**); *n* = 3, for each replicate in per group, three slides were randomly selected, and at least five random fields were examined per cell slide) and phosphor-H3 (Ser 10) ((**J**,**K**); *n* = 3, for each replicate in per group, three slides were randomly selected, and at least five random fields were examined per cell slide), and the of CHOP and GRP78 (**L**,**M**) were detected after mouse GC-1 spg cells were transfected with shNC or shNTE (*n* = 3). Each replicate was the statistical unit, and each dot corresponds to one replicate. Data are presented as mean ± SEM. Statistical significances were determined by using one-way ANOVA followed by Dunnett’s multiple comparisons test. *p* < 0.05 indicates a significant difference between groups.

Neurotoxic target esterase (NTE), as one of the known targets of TOCP, is located in the endoplasmic reticulum of the cell, so we wonder if NTE is involved in TOCP-induced inhibition of proliferation and mitosis of mouse GC-1 spg cells by affecting ER stress. As shown in Figure 5A–F, TOCP dramatically inhibited the activity of NTE in testis tissue and mouse GC-1 spg cells without affecting its expression, unlike shNC, shRNA2, shRNA3, and shRNA4, which all have the effect of reducing NTE (Figure 5G and Figure S3). shRNA2 was used in the subsequent experiments. NTE knockdown decreased the expression of phospho-H3 (Ser 10) and BrdU in mouse GC-1 spg cells (Figure 5H–K), indicating that NTE depletion can inhibit proliferation and mitosis of mouse GC-1 spg cells. Surprisingly, there was no significant change in the protein levels of GRP78 and CHOP in the shNTE-transfected cells (Figure 5L,M). Taken together, the knockdown of NTE inhibits the proliferation and mitosis of mouse GC-1 spg cells.

## 4. Discussion

TOCP is utilized as a flame retardant and plasticizer in industrial products [3], but can accumulate in the brain, testes, kidneys, plasma, and liver, posing a risk to environmental health [4]. Plasticizers can accompany degraded plastics to form microplastics, which have been shown to accumulate in testes and semen and prevent the release of basic nuclear proteins from sperm DNA [33,34]. Previous studies have indicated the male reproductive toxicity of TOCP [13,14,15,16], although the underlying mechanisms remain unclear. Our research revealed abnormalities in the spermatogenic epithelial structure and a reduction in germ cell numbers, along with a decline in sperm count in the cauda epididymis after TOCP exposure. Spermatogenesis is a multifaceted and sequential process that involves the proliferation of spermatogonia, the meiosis of spermatocytes, and the maturation of spermatids into spermatozoa. Any disruption in these stages can result in a lower sperm count. We assessed the quantity of germ cells in the testes using immunohistochemical staining and quantitative PCR. The immunohistochemical analysis indicated a decrease in the number of PCNA- and pH3-positive cells in the testes of mice exposed to TOCP, alongside a notable reduction in the mRNA levels of *c-Kit*. Phospho-histone H3 (Ser 10) is commonly utilized as a marker for mitotic cells [35]. These findings supported that exposure to TOCP may lead to the inhibition of germ cell proliferation within the testes of mice. However, more animals and in vivo experiments are needed to further verify this conclusion.

To explore the reasons behind the inhibition of germ cell proliferation caused by TOCP, we performed in vitro studies using the germ cell line GC-1 spg. Spermatogonial proliferation in the seminiferous tubules occurs through mitotic division [36]. Our findings indicate that TOCP hindered both cell growth and mitosis, as evidenced by immunofluorescent staining for BrdU and pH3 in GC-1 spg cells. Calcium ions are crucial for cell growth, and it was determined that disturbances in Ca^2+^ balance within the endoplasmic reticulum (ER) trigger the activation of stress responses like the unfolded protein response (UPR) [37,38,39,40]. In our previous study, TOCP was shown to activate ER stress in cultured hepatocytes [5]. In this study, exposure to TOCP resulted in an increase in intracellular free Ca^2+^ levels and the upregulation of the ER stress markers CHOP and GRP78. Thapsigargin has been utilized as a model compound to disrupt ER homeostasis and induce UPR [41], while 4-PBA is frequently employed to mitigate ER stress by stabilizing protein structures and aiding in the trafficking of unfolded or misfolded proteins [42]. In our study, 4-PBA was found to rescue the inhibition of cell proliferation and mitosis caused by TOCP, reducing the levels of CHOP and GRP78. These findings imply that TOCP may impair cell growth and mitosis in a manner that is associated with ER stress markers.

The OPIDN hypothesis posits that the disorder arises from the suppression and subsequent aging of NTE within the organism [30]. In vivo, TOCP is processed by cytochrome P450 enzymes into cresyl saligenin phosphate (CBDP) [43,44]. This compound then hinders NTE function and accelerates its aging, contributing to the onset of OPIDN [45,46]. Additionally, CBDP may impede the growth of spermatogonial stem cells through NTE inhibition [16]. We hypothesized that the male reproductive toxicity associated with TOCP could be linked to NTE suppression. In our study, TOCP was found to reduce NTE activity in mouse testes and GC-1 spg cells. In Drosophila melanogaster, mutations in the NTE homolog gene *sws* activate endoplasmic reticulum stress [47]; in our research, we found that levels of the CHOP and GRP78 proteins did not rise in NTE-knockdown GC-1 spg cells. Nevertheless, the knockdown of NTE resulted in decreased cell proliferation and mitotic activity in these cells. These results indicate that TOCP may inhibit cell growth and mitosis through NTE suppression.

Previously, researchers believed that the marginal body weight decreases (17%) in roosters did not contribute to the testicular toxicity induced by TOCP [15]. In our study, we found that TOCP induced significant decreases in mice body weight and epididymis weight, but not testis weight. Previous research found that the testicular changes induced by the IL-8 inhibitor, including seminiferous tubules atrophy and germinal cell degeneration, occurred exclusively in animals with decreased body weight [48]; therefore, we speculate that the toxic effects were considered as be secondary to stress. Additionally, the decrease in epididymis weight may be due to the reduction in sperm count. TOCP can accumulate in testes [4], and its metabolite CBDP has been shown to inhibit spermatogonial stem cell proliferation in vitro [16], which suggests that the testicular damage may be partially secondary to systemic toxicity. At present, this speculation still requires a large amount of data for further verification.

In addition, several limitations should be addressed. A greater number of mouse samples is needed for pathological observation and sperm count statistics. In in vivo experiments, conclusions regarding spermatogonia need to be confirmed using specific markers. The activation of ER stress is primarily mediated by three pathways: PERK/eIF2α/ATF4 signaling, IRE1/XBP1 signaling, and ATF6 activation. It is necessary for further and deeper research to show precisely through which specific pathways TOCP activates ER stress. It should be noted that the NTE knockdown experiments were performed using a single shRNA construct without a rescue experiment. Therefore, although the consistent phenotype supports a role for NTE, we cannot entirely exclude the possibility of off-target effects. The precise molecular mechanisms by which TOCP triggers ER stress remain to be elucidated, as does whether overexpression of NTE can rescue the TOCP-induced proliferation defect.

## 5. Conclusions

In summary, our study found that the TOCP-induced reduction in germ cell proliferation may be mediated through endoplasmic reticulum stress or the suppression of NTE activity, which may lead to decreased sperm count. However, the systemic toxicity of TOCP may be a confounding factor and the mechanistic relationship between ER stress and NTE remains unsolved. These will need to be confirmed in future experiments.

## Figures and Tables

**Table 1:** Primers for qPCR.

	Primer Sequence (5′ to 3′)
*β-actin*-F	CAGCCTTCCTTCTTGGGTAT
*β-actin*-R	TGGCATAGAGGTCTTTACGG
*c-Kit*-F	TGATTGTGCTGGATGATGGATGG
*c-Kit*-R	ATCTGCTCTGCGTCTGTTGGT

Note: Abbreviations: F, forward; R, reverse.

**Table 2:** Primers for shRNA.

	Primer Sequence (5′ to 3′)
shNC-F	CCGGCAACAAGATGAAGAGCACCAACTCGAGTTGGTGCTCTTCATCTTGTTGTTTTTG
shNC-R	AATTCAAAAACAACAAGATGAAGAGCACCAACTCGAGTTGGTGCTCTTCATCTTGTT
shNTE-1F	CCGGGCCTGTATTGGACCTCACATACTCGAGTATGTGAGGTCCAATACAGGCTTTTTT
shNTE-1R	AATTCAAAAAGCCTGTATTGGACCTCACATACTCGAGTATGTGAGGTCCAATACAGG
shNTE-2F	CCGGCCTATGAACGTGGACGGATATCTCGAGATATCCGTCCACGTTCATAGGTTTTTT
shNTE-2R	AATTCAAAAACCTATGAACGTGGACGGATATCTCGAGATATCCGTCCACGTTCATAGG
shNTE-3F	CCGGCCCGCCTTATTCATCTGCTAACTCGAGTTAGCAGATGAATAAGGCGGGTTTTTT
shNTE-3R	AATTCAAAAACCCGCCTTATTCATCTGCTAACTCGAGTTAGCAGATGAATAAGGCGGG
shNTE-4F	CCGGCCTGTTCCTAGACTGGGTTATCTCGAGATAACCCAGTCTAGGAACAGGTTTTTT
shNTE-4R	AATTCAAAAACCTGTTCCTAGACTGGGTTATCTCGAGATAACCCAGTCTAGGAACAGG

Note: Abbreviations: F, forward; R, reverse.

## Data Availability

The original contributions presented in this study are included in the article/Supplementary Materials. Further inquiries can be directed to the corresponding author.

## Supplementary Materials

The following supporting information can be downloaded at https://www.mdpi.com/article/10.3390/toxics14040275/s1, Figure S1: The effects of TOCP on the mice body weight, testis weight and epididymis weight; Figure S2: TOCP reduced sperm count in cauda epididymis; Figure S3: The knockdown efficiency of shRNAs.
