# Supplementary material for: Tri-Ortho-Cresyl Phosphate Inhibits Proliferation of Mouse Germ Cells by Activating Endoplasmic Reticulum Stress and Suppressing NTE Activity
Source: Toxics. 2026 Mar 25;14(4):275. doi: 10.3390/toxics14040275 (PMC13120568; doi:10.3390/toxics14040275)
Supplement: Supplementary file 1 [file toxics-14-00275-s001.zip › toxics-4148226-supplementary.pdf]

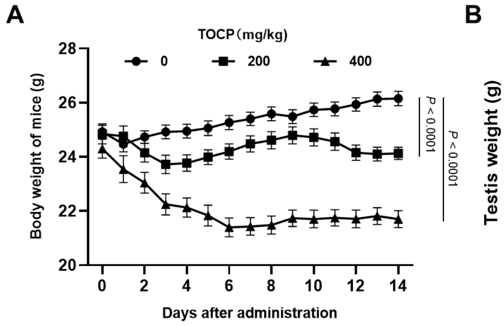

Figure S1. The effects of TOCP on the mice body weight, testis weight and epididymis weight. The male mice were continuously exposed to different doses of TOCP (0 mg/kg, 200 mg/kg, 400 mg/kg) by oral administration for 14 days. (A) The mice body weight. (B) The testis weight. (C) The epididymis weight. Data were expressed as mean ± SEM (n = 10-15). Differences between different groups of body weight were evaluated by two-way ANOVA followed by the Dunnett's multiple comparisons test. Differences between different groups of testis weight and epididymis weight were evaluated by one-way ANOVA followed by the Dunnett's multiple comparisons test. $P < 0.05$ indicates a significant difference between groups.

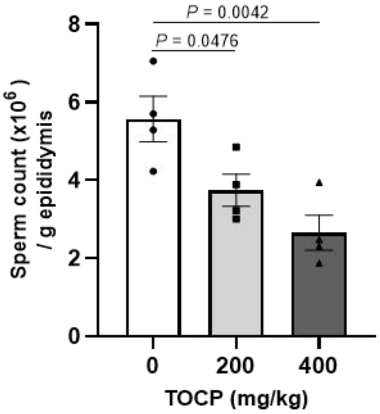

Figure S2. TOCP reduced sperm count in cauda epididymis. The male mice were continuously exposed to different doses of TOCP (0 mg/kg, 200 mg/kg, 400 mg/kg) by oral administration for 14 days. The sperm from cauda epididymis were collected for counting. Data were expressed as mean ± SEM (n = 4), and differences between different groups were evaluated by one-way ANOVA followed by the Dunnett's multiple comparisons test. $P < 0.05$ indicates a significant difference between groups.

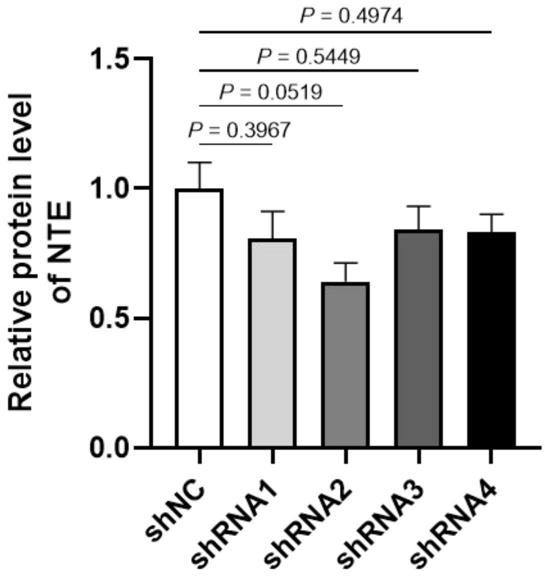

Figure S3. The knockdown efficiency of shRNAs. The shNC and shRNAs lentivirus particles were used for infection of GC-1 spg cells. Stable cells were collected for western blotting. The gray value of NTE was quantified by Image J. Data were expressed as mean ± SEM (n = 3). Differences between different groups of testis weight and epididymis weight were evaluated by one-way ANOVA followed by the Dunnett's multiple comparisons test. $P < 0.05$ indicates a significant difference between groups.
